# Will GLP-1-based therapies reshape the epidemiology of MASLD? An emerging public health perspective

**DOI:** 10.3389/fendo.2026.1981916

**Published:** 2026-10-23

**Authors:** Evelyn Nunes Goulart da Silva Pereira, Karine Lino Rodrigues

**Affiliations:** Laboratory of Clinical and Experimental Pathophysiology, Oswaldo Cruz Institute, Oswaldo Cruz Foundation, Rio de Janeiro, Brazil

**Keywords:** GLP-1 receptor agonists, incretin therapies, MASH, MASLD, obesity, public health, semaglutide, tirzepatide

## Abstract

Metabolic dysfunction-associated steatotic liver disease (MASLD) is the leading cause of chronic liver disease worldwide, driven by increasing obesity and type 2 diabetes mellitus. The expanding use of glucagon-like peptide-1 receptor agonists (GLP-1RAs) raises an epidemiological question: could therapies targeting these metabolic drivers reshape the future burden of chronic liver disease? GLP-1-based therapies reduce body weight and improve metabolic dysfunction, with potential implications for hepatic steatosis and disease progression. The phase 3 ESSENCE trial demonstrated histological benefits of semaglutide in metabolic dysfunction-associated steatohepatitis (MASH) with moderate-to-advanced fibrosis, supporting accelerated or conditional regulatory approvals. However, effects on liver-related clinical outcomes remain unproven. In this Perspective, we examine the evidence and translate it into an explicitly parameterized scenario using a potential impact fraction. Combining published estimates of eligibility, relative risk, treatment persistence, and effectiveness with coverage scenarios of 5%, 10%, and 20%, the model suggests relative reductions of approximately 0.3% to 1.1% in the global incidence of cirrhosis among individuals with prevalent MASLD, at equilibrium (95% uncertainty interval at 20% coverage and intermediate persistence, 0.6% to 1.6%; best-case envelope, 4.6%). These estimates represent testable hypotheses rather than established clinical effects. Effective coverage, defined as coverage multiplied by persistence, is the principal constraint. We identify observations that would refute these expectations and discuss implications for prevention, healthcare systems, and the natural history of MASLD. Population benefits will depend on sustained treatment, accessibility, affordability, and equitable implementation, alongside confirmation that histological improvements translate into fewer liver-related clinical events.

## 1 Introduction

Metabolic dysfunction-associated steatotic liver disease (MASLD) has become the leading chronic liver disease in the modern era, and increasingly represents one of the most prominent hepatic consequences of the global obesity epidemic. Current estimates suggest that nearly one-third of the world’s adult population has some degree of MASLD (1, 2), and projections indicate that this burden will continue to increase in the coming decades. A hierarchical Bayesian forecast based on 245 studies including approximately 2.7 million individuals estimated that the global adult prevalence of fatty liver disease (defined as NAFLD) would reach 55.7% by 2040, assuming that the trend observed in published prevalence studies continues (estimated average annual increase of 2.16% after 2020) (3).

MASLD is no longer confined to high-income countries. Rapid urbanization, nutritional transitions, and declining physical activity have accelerated the rise of obesity-related liver disease across low- and middle-income countries, making MASLD an increasingly important global public health challenge (1, 4). This expanding burden is expected to place substantial pressure on healthcare systems worldwide, reinforcing the need for effective and scalable strategies to prevent disease progression and reduce future healthcare demands (5).

Once considered a relatively benign condition characterized primarily by hepatic fat accumulation, MASLD is now recognized as a complex multisystem disease that may progress to metabolic dysfunction-associated steatohepatitis (MASH), advanced fibrosis, cirrhosis, and hepatocellular carcinoma. It is also closely associated with cardiovascular disease, chronic kidney disease, and increased overall mortality (6, 7).

For many years, therapeutic strategies for MASLD have mainly relied on lifestyle modifications and weight reduction. Although sustained weight loss can substantially improve hepatic steatosis and inflammatory activity, maintaining long-term metabolic improvement in routine clinical practice has proven challenging (8). Consequently, the lack of scalable and durable obesity therapies limits the possibility of meaningfully changing the future burden of MASLD at the population level.

The emergence of glucagon-like peptide-1 receptor agonists (GLP-1RAs) and new incretin-based therapies may fundamentally alter this scenario. Initially developed for the treatment of type 2 diabetes mellitus (T2DM), these agents have achieved levels of weight reduction and metabolic improvement that were previously difficult to attain pharmacologically (9–12). More recently, clinical studies, including a phase 3 histological trial, have shown improvements in hepatic steatosis, aminotransferase levels, and histological features of MASH (10–14).

However, the significance of these therapies may extend beyond the treatment of established liver disease. For the first time, obesity, the main epidemiological driver of MASLD, has become pharmacologically modifiable on a large scale. This raises an increasingly relevant question for hepatology and public health: could the widespread adoption of incretin-based therapies influence the future epidemiology of chronic liver disease?

In this Perspective, we explore the emerging evidence that GLP-1-based therapies could be among the first pharmacological interventions to influence the epidemiology of chronic liver disease at the population level. We also translate this evidence into an explicitly parameterized scenario that yields a falsifiable expectation for the incidence of MASLD-related cirrhosis.

## 2 Obesity, metabolic dysfunction, and MASLD progression

The relationship between obesity and MASLD extends far beyond excess body weight. Adipose tissue dysfunction, especially in visceral depots, promotes chronic low-grade inflammation, increased lipolysis, and ectopic lipid accumulation (15). Excess free fatty acids delivered to the liver contribute to hepatic triglyceride accumulation and lipotoxic injury, ultimately promoting hepatocellular stress and inflammation (16, 17).

Mitochondrial dysfunction and oxidative stress are central components of disease progression. Under sustained metabolic overload, hepatocytes exhibit impaired β-oxidation, increased reactive oxygen species generation, endoplasmic reticulum stress, and activation of inflammatory signaling pathways (16, 18). These mechanisms amplify immune activation and hepatic stellate cell activation, favoring extracellular matrix deposition and fibrosis development (18, 19).

Obesity-related changes in gut microbiota composition and increased intestinal permeability further contribute to hepatic inflammation through gut-liver axis dysregulation (19, 20). The translocation of bacterial-derived inflammatory mediators into the portal circulation perpetuates inflammatory signaling and metabolic dysfunction.

However, obesity alone does not fully explain the disease heterogeneity. Several studies suggest that body composition and fat distribution may be more relevant than body mass index itself (21–23). Individuals with lean MASLD often exhibit increased visceral adiposity, sarcopenia, and adverse metabolic profiles despite normal body weight (22, 23).

Sarcopenia has emerged as another important determinant of disease severity and fibrosis progression. Reduced skeletal muscle mass impairs peripheral glucose utilization and substrate buffering capacity, increasing hepatic exposure to glucose and free fatty acids (21–23). Altered muscle-liver signaling may also contribute directly to hepatic inflammation and metabolic dysregulation.

The fibrosis stage remains the strongest predictor of liver-related and overall mortality in MASLD (22–24). Consequently, interventions that reduce obesity, improve insulin resistance, and attenuate fibrosis progression may substantially influence long-term clinical outcomes.

## 3 The incretin era and the transformation of obesity treatment

For decades, pharmacological treatments for obesity have shown limited efficacy and poor long-term sustainability. Lifestyle interventions remain fundamental, but are often limited by poor adherence and compensatory metabolic adaptations that favor weight regain (8). Bariatric surgery remains highly effective, although its applicability is inherently limited by cost, infrastructure, and patient eligibility.

The development of GLP-1RAs has transformed the therapeutic landscape. Originally introduced for T2DM treatment, GLP-1RAs have demonstrated substantial effects on appetite regulation, satiety, delayed gastric emptying, and body weight reduction (9).

Semaglutide and tirzepatide now achieve levels of weight loss approaching those previously observed mainly after metabolic surgery (9, 11). In STEP 1, semaglutide 2.4 mg once weekly produced a mean body weight reduction of 14.9% at 68 weeks, versus 2.4% with placebo (estimated treatment difference, −12.4 percentage points; 95% CI, −13.4 to −11.5) (9). In SURMOUNT-1, tirzepatide produced mean reductions of 15.0%, 19.5%, and 20.9% with the 5-, 10-, and 15-mg doses, respectively, at 72 weeks, versus 3.1% with placebo (11). Both trials also reported improvements in insulin sensitivity, glycemic control, blood pressure, and overall cardiometabolic risk (9, 11). In the STEP 1–3 trials, semaglutide 2.4 mg reduced C-reactive protein, a marker of systemic inflammation, by 39% to 48% relative to placebo at week 68 (25).

Importantly, these therapies also have documented hepatic effects. In a phase 2 trial in patients with MASH, semaglutide increased the rate of steatohepatitis resolution compared with placebo, without a significant difference in fibrosis improvement (10). The pivotal phase 3 ESSENCE trial subsequently randomized 1,197 patients with biopsy-defined MASH and fibrosis stage F2–F3 to semaglutide 2.4 mg once weekly or placebo (14). In the first 800 patients assessed at week 72, resolution of steatohepatitis without worsening of fibrosis occurred in 62.9% versus 34.3% (estimated difference, 28.7 percentage points; 95% CI, 21.1 to 36.2), and fibrosis improvement without worsening of steatohepatitis in 36.8% versus 22.4% (estimated difference, 14.4 percentage points; 95% CI, 7.5 to 21.3). Mean body weight changed by −10.5% versus −2.0% (14). Tirzepatide also improved MASH resolution and fibrosis in the phase 2 SYNERGY-NASH trial (26). In the earlier, exploratory phase 2 LEAN trial (n = 52), liraglutide was associated with resolution of steatohepatitis; less frequent fibrosis progression was an underpowered secondary finding based on few paired biopsies (12).

These results have three limitations relevant to population-level inference: the endpoints are histological surrogates, and their translation into fewer liver-related clinical events awaits the outcomes phase of the trial (part 2, 240 weeks); the absolute fibrosis benefit is modest (approximately seven patients treated for 72 weeks per additional patient with fibrosis improvement); and patients with cirrhosis were excluded and 67.5% of participants were White, which limits extrapolation to the populations in which MASLD is growing fastest (14).

On this basis, semaglutide 2.4 mg received accelerated approval from the US Food and Drug Administration in August 2025 (27), a conditional marketing authorisation in the European Union in March 2026, under the brand name Kayshild (28), and approval from the Brazilian Health Regulatory Agency (Anvisa) in December 2025 (29), in each case for adults with non-cirrhotic MASH and F2–F3 fibrosis; the US and EU authorisations are contingent on confirmation of clinical benefit. These decisions rest on surrogate endpoints and do not cover the far larger populations with steatosis or early fibrosis in whom population-level prevention would have to occur.

Beyond isolated GLP-1 receptor agonism, newer dual and triple incretin agonists may further enhance these effects through the combined modulation of the GLP-1, glucose-dependent insulinotropic polypeptide, and glucagon receptor pathways, as suggested by phase 2 trials of tirzepatide, survodutide, and retatrutide (26, 30, 31). These advances have generated considerable interest in the role of incretin-based therapies for managing metabolic liver disease. However, the broader significance of these therapies may extend beyond individual metabolic improvement.

## 4 From obesity treatment to population-level liver disease prevention

Beyond their efficacy, GLP-1-based therapies have rapidly become part of routine clinical practice worldwide. What began as a diabetes-focused therapeutic strategy has rapidly evolved into a large-scale intervention targeting obesity and metabolic disease more broadly (9–12, 32–34).

This therapeutic effect contributes to a significant shift in the perception of obesity. Historically treated primarily as a behavioral issue, obesity is now increasingly recognized as a chronic, relapsing disease requiring long-term, multifactorial management; this framing, already present in the 2016 AACE/ACE guidelines (34), was formalized by the 2025 Lancet Commission on clinical obesity (33). Guidelines issued after the approval of semaglutide 2.4 mg for obesity support long-term pharmacotherapy as chronic disease management rather than as a temporary adjunct (35, 36), and the 2025 framework of the European Association for the Study of Obesity positions semaglutide and tirzepatide as first-line options (36).

The introduction of semaglutide, tirzepatide, and related agents into obesity medicine, cardiovascular prevention programs, endocrinology practice, and increasingly, into primary care reflects a broader transformation in the management of chronic metabolic diseases. In the United States, uptake among eligible adults reached a median of 6.5% across states (37).

This shift may have profound implications from a hepatological perspective. Because obesity, insulin resistance, and visceral adiposity are the principal upstream drivers of MASLD, the widespread use of highly effective anti-obesity therapies raises the hypothesis that future incidence rates of metabolic liver disease could decline. **Table 1** separates the effects of these agents that have been established in randomized trials from the hepatic and population-level consequences that remain inferential or untested.

**Table 1:** Hypothesis framework linking established effects of GLP-1-based therapies to plausible hepatic consequences and untested population-level implications for MASLD.

GLP-1 effect	Potential hepatic consequence	Population-level implication (hypothesis)	Evidence type	References
Sustained weight reduction	Decreased hepatic steatosis	Hypothesis: lower future incidence of MASLD	Weight loss: RCT, surrogate endpoint. Steatosis: RCT, histological endpoint. Population effect: hypothesis, untested	(8, 9, 11, 14)
Reduction in visceral adiposity	Possible reduction in hepatic lipid flux and lipotoxicity	Hypothesis: slower growth of obesity-related liver disease burden	Visceral fat: RCT, imaging substudy (surrogate). Hepatic consequence: mechanistic inference. Population effect: hypothesis, untested	(9); mechanistic (15, 16):
Improved insulin sensitivity	Possible attenuation of hepatic metabolic stress	Hypothesis: delayed progression to MASH	Insulin sensitivity: RCT, surrogate endpoint. Hepatic consequence: mechanistic inference. Population effect: hypothesis, untested	(9, 11); mechanistic (17):
Reduction in C-reactive protein	Possible attenuation of fibrogenic signaling (not tested for GLP-1-based therapies)	Hypothesis: lower long-term fibrosis burden	CRP: RCT, secondary endpoint. Hepatic consequence: mechanistic inference from reviews that did not evaluate GLP-1-based therapies. Population effect: hypothesis, untested	(25); mechanistic (18, 19):
Improvement in cardiometabolic risk profile	Reduced multisystem metabolic dysfunction associated with MASLD	Fewer cardiovascular events (shown in obesity with established cardiovascular disease, not specifically in MASLD); hypothesis: lower liver-related mortality	Cardiovascular events: RCT, hard clinical endpoint. Liver-related mortality: hypothesis, untested	(6, 7, 38)
Histological improvement in MASH	Resolution of steatohepatitis; fibrosis improvement in a minority (+14.4 percentage points over placebo at 72 weeks in F2–F3)	Hypothesis: decline in cirrhosis incidence; liver-related clinical outcomes pending (ESSENCE part 2)	RCT, histological endpoint (phase 3 for semaglutide; phase 2 for tirzepatide and liraglutide). Clinical outcomes: not yet reported	(10, 12, 14, 26); prognostic value of fibrosis (24):
Reduction in liver fat content	Improvement in hepatic metabolic homeostasis	Hypothesis: lower demand for hepatology-related healthcare resources	Liver fat: RCT, imaging/histological surrogate. Resource use: hypothesis, untested	(13, 14, 26); burden (4):
Earlier metabolic intervention before advanced fibrosis	Possible prevention of progressive liver injury	Hypothesis: reduced future liver transplantation demand	Hypothesis, untested (no trials with liver outcomes in F0–F1 or at-risk populations)	(5, 8, 24)
Expansion of obesity pharmacotherapy into primary care	Broader treatment of metabolic risk factors	Hypothesis: population-level modification of chronic liver disease trajectories	Guideline recommendations (evidence review/expert consensus). Population effect: hypothesis, untested	(32, 33, 35, 36)
Long-term chronic obesity management	Sustained control of metabolic dysfunction, conditional on persistence	Hypothesis: attenuation of future HCC burden and liver-related mortality	Persistence: observational, real-world data (most patients discontinue within 1 year). Population effect: hypothesis, untested	(1, 5, 39, 40)

GLP-1, glucagon-like peptide-1; HCC, hepatocellular carcinoma; MASH, metabolic dysfunction-associated steatohepatitis; MASLD, metabolic dysfunction-associated steatotic liver disease. CRP, C-reactive protein; RCT, randomized controlled trial. Evidence type: RCT, hard clinical endpoint; RCT, histological endpoint; RCT, surrogate endpoint (body weight, imaging, or biomarkers); observational; mechanistic inference (biological plausibility derived from pathophysiology reviews that did not evaluate GLP-1-based therapies); hypothesis, untested. All entries in the population-level column are hypotheses; none has been established. References for mechanistic inference are listed separately from references that evaluated GLP-1-based therapies.

This possibility marks an important departure from traditional hepatology. Historically, most liver-directed therapies have focused on established diseases, often after fibrosis progression or cirrhosis had already developed. In contrast, incretin-based therapies intervene much earlier in the metabolic cascade, potentially modifying the systemic drivers involved in disease initiation long before advanced liver injury becomes clinically apparent.

Epidemiological implications are especially relevant in light of current global trends. Obesity rates continue to rise worldwide, particularly among younger populations and in low- and middle-income countries (39). At the same time, modeling studies predict substantial increases in MASLD-related cirrhosis, liver failure, hepatocellular carcinoma, and liver transplantation over the coming decades if current trends persist (1, 5).

In this context, the rapid spread of highly effective anti-obesity therapies introduces a possibility that until recently seemed unrealistic: reducing the future burden of chronic liver disease through large-scale modification of metabolic risk itself. Whether this possibility is realistic, and of what magnitude, is an empirical question that can be framed quantitatively (Section 5.1).

## 5 Could GLP-1-based therapies modify the future epidemiology of MASLD?

The relationship between sustained weight reduction and improvement in MASLD is well established. Weight loss improves hepatic steatosis, reduces inflammatory activity, improves insulin sensitivity, and may attenuate fibrosis progression (8). Consequently, therapies capable of producing substantial and durable metabolic improvements may reduce the number of individuals who develop MASLD or progress along its spectrum.

At the population level, the expected chain of effects is as follows. If incretin-based therapies become broadly accessible, future reductions in obesity prevalence may translate into a lower incidence of hepatic steatosis, delayed progression to MASH, reduced fibrosis burden, lower hepatocellular carcinoma incidence, decreased liver transplantation demand, and ultimately, reduced liver-related mortality. Importantly, these changes are likely to emerge gradually over decades rather than years, reflecting the relatively slow progression of fibrosis and cirrhosis.

The public health implications of this shift are significant. MASLD is already a major source of healthcare expenditure due to cirrhosis management, transplantation, hepatocellular carcinoma surveillance, and disability (4, 5). Therapies that reduce the obesity-driven disease burden could substantially impact healthcare systems worldwide.

However, important limitations should be considered. Access to incretin-based therapies remains highly unequal across countries and healthcare systems. Populations experiencing the fastest increase in obesity and MASLD often have the least access to advanced pharmacotherapy. High costs, reimbursement barriers, and limited healthcare infrastructure may restrict their broader implementation.

In addition, long-term durability is not fully understood. Weight regain after treatment discontinuation appears to be common, suggesting that sustained therapy is necessary to maintain the long-term metabolic benefits. Persistence in routine care is limited: in a cohort of 125,474 US adults with overweight or obesity who initiated a GLP-1RA, 46.5% of those with and 64.8% of those without T2DM discontinued treatment within 1 year (40).

Although incretin-based therapies have consistently improved steatosis and metabolic dysfunction, their effects on advanced fibrosis remain less pronounced. In ESSENCE, the absolute benefit on fibrosis improvement (14.4 percentage points) was approximately half that on steatohepatitis resolution (28.7 percentage points) (14). Whether these agents can substantially reduce the incidence of cirrhosis at the population level is therefore an empirical question, addressed quantitatively in Section 5.1.

### 5.1 An illustrative, testable scenario

To convert the central question into a testable proposition, we estimated the potential impact fraction (PIF), the standard epidemiological measure of the proportional change in disease burden after a change in exposure (41, 42) (**Table 2**). For an intervention delivered to eligible individuals with MASLD, PIF = Ew × C × P × R, where C is coverage among eligible individuals, P is persistence among initiators, R is the relative reduction in major adverse liver outcomes during treatment, derived from an observational target trial emulation in patients with T2DM and used as a proxy for the effect on incident cirrhosis, and Ew is the share of outcome events arising in eligible individuals, weighted by their relative risk (RR): Ew = e·RR/[(1 − e) + e·RR]. Eligibility was approximated by non-lean status. Values of e, RR, P, and R were taken from published data (22, 40, 43, 44). As in a published microsimulation of GLP-1RA coverage for obesity (45), C was examined at 5%, 10%, and 20%, which bracket currently observed uptake (37). The PIF was applied to the expected global number of incident MASLD-related cirrhosis cases, estimated as the product of the Global Burden of Disease 2021 number of prevalent MASLD cases, 1.27 billion (46), and the pooled incidence of cirrhosis in MASLD cohorts, 10.85 per 1,000 person-years (95% CI 6.65–15.06) (47), approximately 13.8 million per year; because the pooled cohorts were clinically enriched, this is an upper bound. The estimate therefore covers the treatment effect in prevalent MASLD; it does not incorporate prevention of incident MASLD through reduced obesity, and the total population effect would be the sum of both pathways. It also represents the equilibrium effect, reached only after a latency reflecting fibrosis progression; intermediate-term reductions would be smaller.

**Table 2:** Potential impact fraction (PIF) of incretin-based therapies on the global incidence of MASLD-related cirrhosis under coverage scenarios of 5%, 10%, and 20%.

A. Parameter	Definition	Low	Intermediate	High	Source of values
e	Proportion of the MASLD population eligible for incretin-based therapy (non-lean)	0.770	0.808	0.841	1 − lean proportion of the global MASLD population, 19.2% (95% CI 15.9–23.0) (22)
RR	Relative risk of decompensated cirrhosis or hepatocellular carcinoma, eligible vs lean (proxy for cirrhosis)	0.77	1.23	2.00	Inverse of lean vs non-lean RR 0.81 (95% CI 0.50–1.30; not statistically significant) (44)
Ew	Share of cirrhosis cases arising in eligible individuals, e·RR/[(1 − e) + e·RR]	0.72	0.84	0.91	Calculated
P	Persistence among initiators	0.278	0.352	0.535	2-year persistence, all patients (longer horizon); 1-year persistence without and with T2DM (40)
R	Relative reduction in major adverse liver outcomes during treatment (1 − HR)	0.10	0.19	0.47	Semaglutide vs DPP-4 inhibitors, HR 0.81 (95% CI 0.72–0.90), upper limit and point estimate; tirzepatide vs DPP-4 inhibitors, HR 0.53 (43)
Incident cirrhosis	Expected global incident MASLD-related cirrhosis per year: prevalent MASLD (1,267.8 million) × incidence rate	8.4 million	13.8 million	19.1 million	GBD 2021 prevalence (46); pooled incidence of cirrhosis 10.85 per 1,000 person-years (95% CI 6.65–15.06) (47)
B. Coverage (C)	Basis	C × P (intermediate P)	PIF, intermediate (envelope)	Cases averted per year, intermediate	Cases averted per year, envelope
5%	Low uptake scenario of a GLP-1RA microsimulation (45); observed uptake 2.8%–9.8% (37)	1.8%	0.28% (0.10%–1.15%)	≈39,000	≈8,000–219,000
10%	Base-case uptake of the same microsimulation (45)	3.5%	0.56% (0.20%–2.30%)	≈77,000	≈17,000–439,000
20%	High uptake scenario of the same microsimulation (45)	7.0%	1.12% (0.40%–4.59%)	≈154,000	≈34,000–877,000

DPP-4, dipeptidyl peptidase-4; GBD, Global Burden of Disease; GLP-1RA, glucagon-like peptide-1 receptor agonist; HR, hazard ratio; MetALD, metabolic dysfunction and alcohol-associated liver disease; RR, relative risk; T2DM, type 2 diabetes mellitus. PIF = Ew × C × P × R, derived from the relative-risk-shift form of the potential impact fraction (41, 42); PIF values are relative reductions, not absolute percentage points. Ranges combine all low or all high parameter values, including the incidence rate; they are best-case/worst-case envelopes with a very low joint probability, not uncertainty intervals. A probabilistic sensitivity analysis (100,000 Monte Carlo draws; beta distribution for the lean proportion, log-normal distributions for RR and for the semaglutide HR, and normal distribution for the incidence rate) was run separately for each persistence value, because the three values of P reflect different horizons and populations rather than a single uncertain quantity. With intermediate persistence, the median PIF (95% uncertainty interval) was 0.28% (0.14%–0.41%), 0.56% (0.28%–0.82%), and 1.11% (0.55%–1.64%) at 5%, 10%, and 20% coverage, or approximately 37,000 (16,000–64,000), 75,000 (33,000–129,000), and 149,000 (65,000–257,000) cases averted per year; at 20% coverage, it was 0.88% (0.44%–1.29%) with low and 1.69% (0.84%–2.49%) with high persistence. In one-way sensitivity analyses at 10% coverage, R (0.30%–1.39%, the upper value reflecting the tirzepatide estimate) and P (0.44%–0.85%) accounted for most of the width of the envelope, whereas e (0.54%–0.58%) and RR (0.51%–0.60%) had little influence. The model assumes independent parameters, a steady state, and no residual benefit after discontinuation; the PIF is the effect at equilibrium, reached only after a latency reflecting fibrosis progression, and intermediate-term reductions would be smaller. Two biases operate in opposite directions. The pooled incidence of cirrhosis comes from cohorts that were clinically enriched (58.9% with NASH at baseline) (47), so the absolute numbers are upper bounds; however, enrichment does not affect the percentage PIF, because numerator and denominator scale together, so the proportional result is more robust than the absolute figures. Conversely, the model quantifies the treatment effect in prevalent MASLD and omits the prevention of incident MASLD through reduced obesity, which biases the total population effect downward. The GBD category of incident MASLD-related chronic liver disease and cirrhosis (48.3 million cases in 2021) was not used, because it does not separate cirrhosis from earlier stages (46). Limitations: eligibility is approximated by non-lean status, which places lean MASLD (19.2%) outside any obesity-anchored strategy; e is derived from prevalence; MetALD, which has different progression dynamics and responsiveness to metabolic intervention, is not included in the denominator (6); the RR for decompensated cirrhosis or hepatocellular carcinoma is used as a proxy for cirrhosis, and its source estimate did not reach statistical significance, which limits the weight Ew can carry (44); P combines time horizons (the low value is 2-year persistence, the intermediate and high values 1-year persistence), ignores reinitiation, which occurred within 1 year in 36.3% to 47.3% of those who discontinued (40), and is unknown over the 10- to 20-year horizon relevant to cirrhosis; R comes from observational data in people with T2DM over 2 years (43), and 1 − HR treats a hazard ratio as a risk ratio, which tends to overstate the relative risk reduction when outcomes are common or follow-up is long (with a 2-year risk of 1% to 5%, 1 − RR would be 0.186 to 0.189 rather than 0.19); and GLP-1RA-associated loss of lean mass (9), if it modifies progression risk, would reduce R, particularly in older adults, who carry most of the advanced fibrosis burden. Fibrosis progression was not modeled, because pooled incidence data derive from only five small cohorts (47). No comparable coverage data were found for low- and middle-income countries.

With intermediate parameter values, the PIF for incident MASLD-related cirrhosis, a relative reduction, is 0.28%, 0.56%, and 1.1% at 5%, 10%, and 20% coverage, corresponding to approximately 39,000, 77,000, and 154,000 cases averted per year. In a probabilistic sensitivity analysis by persistence scenario, 95% uncertainty intervals with intermediate persistence were 0.14%–0.41%, 0.28%–0.82%, and 0.55%–1.64%; the wider ranges in **Table 2**, reaching 4.6% at 20% coverage, are best-case/worst-case envelopes rather than uncertainty intervals.

Three conclusions follow. First, within observed or modeled coverage, the expected reduction in incident cirrhosis is about 1% or less with intermediate parameters, below 2.5% within any uncertainty interval, and below 5% even in the best-case envelope. Second, effective coverage (C × P), rather than drug efficacy, is the principal constraint: at 20% coverage and intermediate persistence, 7% of eligible individuals receive sustained treatment. Persistence is the least certain term: available data cover 1 to 2 years, whereas cirrhosis develops over decades, and persistence over a 10- to 20-year horizon is unknown and unlikely to be higher. Because 19.2% of individuals with MASLD are lean, an obesity-anchored strategy has a structural ceiling on its population effect. Third, the expectation is falsifiable. The relevant observation is the age-standardized incidence of cirrhosis among individuals with recorded MASLD in population-based registries that link prescriptions to hospital diagnoses, such as the Nordic national registries. A relative decline greater than 5% where coverage of at least 20% has been sustained for 10 to 20 years, compared by difference-in-differences with low-coverage regions or periods and adjusted for trends in obesity and diabetes, would indicate that R is underestimated; no decline under such coverage would indicate overestimation. Coverage data for low- and middle-income countries, including Brazil, and the outcomes phase of ESSENCE (14) are research priorities.

## 6 Discussion

The future of MASLD management will likely depend on a shift from liver-centered treatment strategies to broader approaches focused on metabolic disease prevention. The recognition that adipose tissue dysfunction, visceral adiposity, sarcopenia, and systemic inflammation are major disease drivers highlights the need for integrated therapeutic models. Phenotype-guided approaches that incorporate body composition, genetic susceptibility, metabolic risk, and fibrosis assessments may become increasingly important (21).

In this context, incretin-based therapies, which act on upstream metabolic drivers rather than on established liver disease as liver-directed agents such as resmetirom do (48), are among the first widely deployed pharmacological tools for which an effect on the epidemiology of chronic liver disease can be plausibly hypothesized and quantitatively tested. Our scenario indicates that, at current levels of treatment coverage and persistence, this effect is expected to be small, and that effective coverage, rather than drug efficacy, is the principal constraint. Their greatest impact may ultimately occur before fibrosis develops, by preventing obesity-driven metabolic dysfunction. Whether this potential is realized depends on factors such as accessibility, affordability, long-term adherence, sustained efficacy, and effective healthcare policy implementation.

Whether the incretin era will ultimately lead to measurable reductions in cirrhosis, hepatocellular carcinoma, liver transplantation, and liver-related mortality remains unproven; the scenario presented here defines the magnitude of change that would be expected and, therefore, what future surveillance data would need to show. The incretin era has changed the conversation about MASLD: hepatology is now considering not only how to treat steatohepatitis, but also whether the future burden of chronic liver disease itself can be modified.

## Data Availability

The raw data supporting the conclusions of this article will be made available by the authors, without undue reservation.
